# Big data evidence of the impact of COVID-19 hospitalizations on mortality rates of non-COVID-19 critically ill patients

**DOI:** 10.1038/s41598-023-40727-z

**Published:** 2023-08-21

**Authors:** Bruno Wichmann, Roberta Moreira Wichmann

**Affiliations:** 1 Department of Resource Economics & Environmental Sociology, College of Natural and Applied Sciences, University of Alberta, 503 General Services Building, Edmonton, AB T6G-2H1 Canada; 2 World Bank, Brasília, Brazil; 3 Brazilian Institute of Education, Development and Research–IDP, Brasília, Brazil

**Keywords:** Diseases, Health care, Risk factors

## Abstract

The COVID-19 virus caused a global pandemic leading to a swift policy response. While this response was designed to prevent the spread of the virus and support those with COVID-19, there is growing evidence regarding measurable impacts on non-COVID-19 patients. The paper uses a large dataset from administrative records of the Brazilian public health system (SUS) to estimate pandemic spillover effects in critically ill health care delivery, i.e. the additional mortality risk that COVID-19 ICU hospitalizations generate on non-COVID-19 patients receiving intensive care. The data contain the universe of ICU hospitalizations in SUS from February 26, 2020 to December 31, 2021. Spillover estimates are obtained from high-dimensional fixed effects regression models that control for a number of unobservable confounders. Our findings indicate that, on average, the pandemic increased the mortality risk of non-COVID-19 ICU patients by 1.296 percentage points, 95% CI 1.145–1.448. The spillover mortality risk is larger for non-COVID patients receiving intensive care due to diseases of the respiratory system, diseases of the skin and subcutaneous tissue, and infectious and parasitic diseases. As of July 2023, the WHO reports more than 6.9 million global deaths due to COVID-19 infection. However, our estimates of spillover effects suggest that the pandemic’s total death toll is much higher.

## Introduction

In December 2019, the severe acute respiratory syndrome coronavirus 2 (SARS-CoV-2) emerged from China and quickly spread around the world causing a global pandemic. The virus’ high contagion rates, its ability to mutate, and the serious morbidity and mortality associated with its disease (COVID-19), especially in vulnerable cohorts, triggered an unprecedented policy response.

The policy response arises from two angles. First, from a macro (national public policy) perspective, many countries implemented port-of-entry restrictions, school closures, curfews and lockdowns, and restrictions on nonessential businesses^1^. While these measures may slow the spread of SARS-CoV-2, recent work uncovers their large economic impacts^2^. This generates concerns due to the feedback from the economic downturn to health outcomes, raising questions of what are the health consequences to the general population of the swift COVID-19 public policies^3,4^.

Second, from a micro perspective, the pandemic forced hospitals and healthcare facilities to develop protocols to cope with the increased demand for health care. Limited resources and the prospect of a flood of COVID-19 patients forced hospitals around the world to make trade-offs. In addition, many patients developed a sense of hesitation to seek health care as hospitals and clinics are perceived as places of high infection risk^5^. This is especially worrisome as recent evidence suggest that more vulnerable older patients delayed seeking health care during the pandemic^6^. While digital technologies can mitigate this issue, they can also contribute to health care inequalities as digital medicine is disproportionately available across patients of varying income levels^7^.

The pandemic has the potential to create challenges associated with both the demand and supply of health, e.g. late diagnosis and resource scarcity, respectively. As a result, there is a growing need for studies that go beyond health outcomes of patients infected with SARS-CoV-2 and examine outcomes of the general patient population in an attempt to identify possible spillover effects. Of particular importance is the impact of the pandemic environment on a set of patients whose vulnerability increases with the intensity of local SARS-CoV-2 outbreaks: non-COVID-19 patients in intensive care units. There have been many reports about the devastating impact of the pandemic on those infected with SARS-CoV-2^8–10^, including disproportionate impacts on minorities^11^. In addition, a growing literature reports associations between the COVID-19 pandemic and health outcomes of non-COVID-19 patients^12–15^.

The goal of the paper is to test for spillover effects, i.e. a decrease in health outcomes of non-COVID-19 patients as a result of the intensification of the COVID-19 pandemic. We employ big data and high-dimensional panel data techniques to provide an estimate of the impact of the COVID-19 pandemic on non-COVID-19 ICU patients. We exploit electronic administrative health records from the largest public health care system in the world, the Brazilian Unified Health System–hereafter SUS (refer to the Supplemental Materials for more information regarding SUS and the institutional setting). In summary, we estimate the additional mortality risk that COVID-19 ICU hospitalization generates to patients that are not identified as carrying the SARS-CoV‑2 virus but receive intensive care in a hospital-week with ICU patients being treated for COVID-19.

## Results

**Figure 1:**
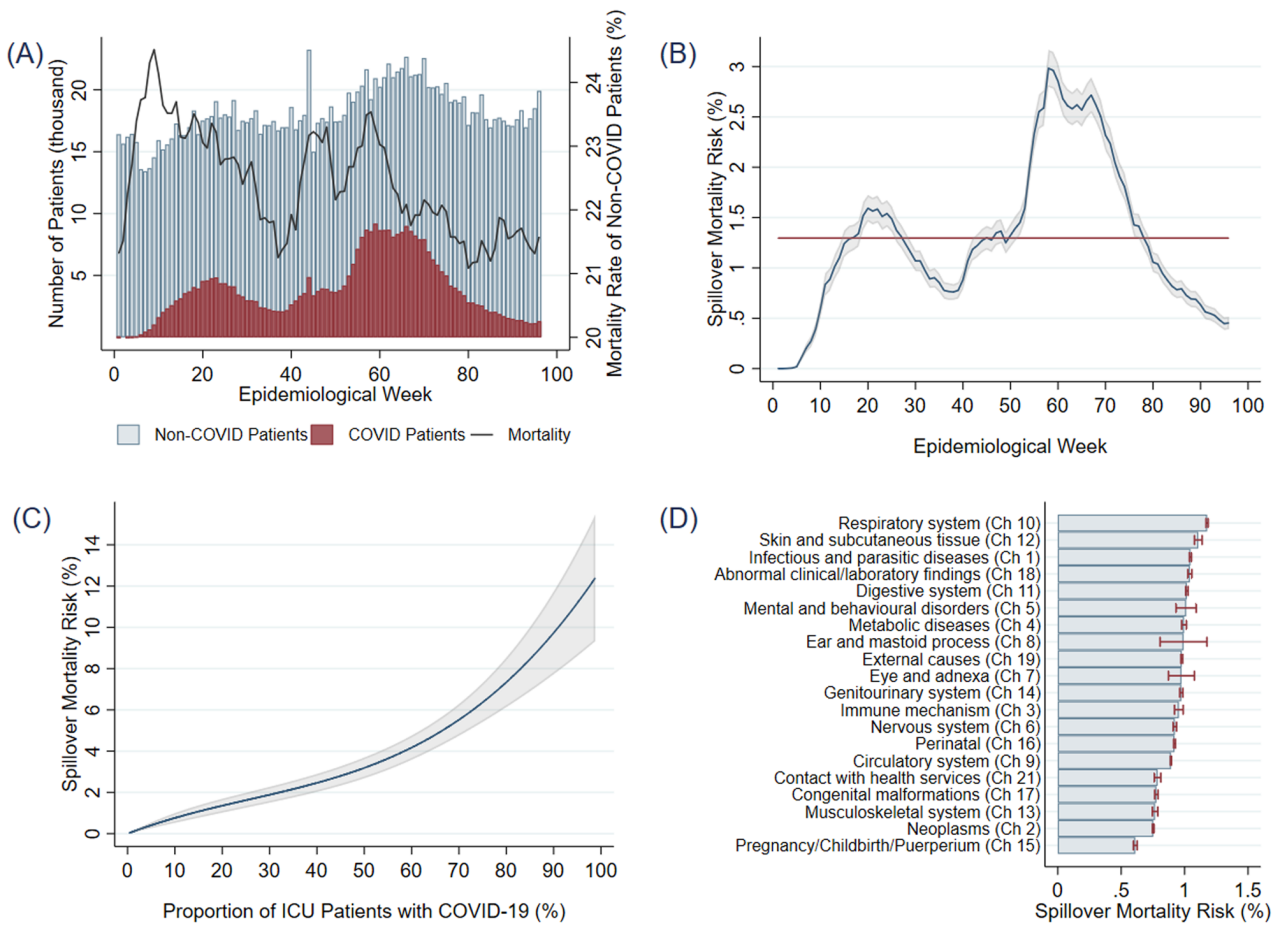
(**A**) Evolution over 96 epidemiological weeks (from February 26, 2020 to December 31, 2021) of the number of ICU patients, by COVID diagnosis, and (7 day) moving average of the mortality rate of non-COVID-19 patients. (**B**) Estimates of the spillover mortality risk, i.e. $\widehat{f\left({P}_{jt}\right)}$ as estimated by model (1), and its 95% confidence interval, aggregated (averaged) by epidemiological week. The horizontal line represents the overall average of 1.3%. (**C**) The spillover mortality risk, $\widehat{f\left({P}_{jt}\right)}$, as a function of the proportion of ICU patients diagnosed with COVID-19, ${P}_{jt}$. (**D**) Average spillover mortality risks and 95% confidence intervals, by chapters of the ICD-10 disease classification.

The results are summarized in Fig. 1 (full estimates of model (1) are available in the supplemental materials, Table 2). Panel (A) shows the evolution of number of ICU patients by COVID-19 diagnosis. The figure also shows the 7-day moving average of the mortality rate of non-COVID-19 ICU patients. The panel suggests a positive correlation between health outcomes of non-COVID-19 ICU patients and the number of COVID-19 patients (correlation coefficient is 0.1287). Our goal is to move beyond correlations and estimate the parameters of the high-dimensionality model (1) that improves identification of spillover effects by controlling for unobserved heterogeneity in the data.

Panel (B) plots model 1’s estimates of the spillover mortality risks, i.e. the fraction on the mortality risk of non-COVID-19 ICU patients that is due to the presence of COVID-19 patients during their hospitalization. The results show that, after controlling for six important determinants of ICU outcomes (see below), spillover mortality risk peaked at approximately 3% just before epidemiological week 60 (April 16, 2021–April 22, 2021). This means that, holding constant:

1. health demand factors from socioeconomic local conditions of the residence of patients^16–18^,
2. health supply factors associated with hospital fixed characteristics^19^,
3. common shocks across the system^20,21^,
4. the reason for ICU hospitalization (diagnosis)^22,23^,
5. time-varying (non-linear) sex and ages effects^24^, and
6. time-varying hospital capacity (number of ICU patients)^25^,

the mortality risk of a non-COVID-19 patient receiving intensive care during the 60th epidemiological week is three percentage points higher than baseline mortality, i.e. the mortality of the proxy patient (as per controls above) had the patient been hospitalized in a hospital-week with no COVID-19 patients. Over the 96 epidemiological weeks considered, the spillover mortality risk averaged at 1.3% (red horizontal line).

Panel (C) slices the data in a different way by plotting the spillover mortality risk as a function of the proportion of ICU patients diagnosed with COVID-19, ${P}_{jt}$. Our model allows us to estimate the spillover mortality risk non-linearly. The figure shows that spillover mortality risk is fairly linear up to 50% of COVID-19 patients, beyond which risk increases at an increasing rate. For low levels of ICU utilization by COVID-19 patients $({P}_{jt}<20)$, the additional mortality risk generated to non-COVID-19 patients is 0.698%. For high levels of service to COVID-19 patients $({P}_{jt}>80)$, average spillover mortality risk increases to 9.680%. We note that the 95% confidence interval becomes wider as the proportion of COVID-19 patients increases beyond 80% reflecting the fact that sample sizes under those circumstances decrease.

The mortality risk ratio for a non-COVID-19 patient receiving intensive care in a hospital-week where 75% of patients are COVID-19 patients, relative to receiving care in a hospital-week where 25% of patients have COVID-19, is:

$$\frac{Risk\,\left(75\%\right)}{Risk\,(25\%)}=3.92 \,(95\%\,\mathrm{CI}\,3.08{-}4.77)$$

The nonlinearity of effects is apparent as this ratio increases (more than) three times when we compare risks at ${P}_{jt}=90\%$ vs ${P}_{jt}=10\%$, i.e.

$$\frac{Risk\,\left(90\%\right)}{Risk\,(10\%)}=12.77\, (95\%\,\mathrm{CI}\,9.10{-}16.44)$$

Finally, panel (D) shows average spillover risks, along with their 95% confidence intervals, by chapter of ICD-10 diagnosis. Specifically, this panel uses patient-level spillover predictions from model (1) and aggregates them by disease chapter. In general, the figure shows a lack of heterogeneity in how the COVID-19 pandemic affected non-COVID-19 ICU patients. Average spillover risk is higher for patients receiving intensive care for diseases classified under chapter 10 of the ICD-10, namely ‘diseases of the respiratory system’. For these non-COVID-19 patients, receiving intensive care in a hospital-week where intensive care is also provided to COVID-19 patients increases mortality risk by an average of 1.18%, with 95% C.I. 1.17–1.19%. The second highest predicted spillover effect is that affecting patients receiving intensive care for ‘diseases of the skin and subcutaneous tissue’ (Ch. 12), where the additional risk from the presence COVID-19 patients is 1.11%, with 95% C.I. 1.08–1.14%. As the figure shows, many other diseases are subject to spillover risks hovering around 1%, from the third highest ‘infectious and parasitic diseases’ (1.05%) to the fourteenth highest ‘certain conditions originating in the perinatal period’ (0.92%). The lowest spillover effect is identified in ICU patients being treated for conditions in chapter 15, ‘pregnancy, childbirth and the puerperium’ (0.61%).

**Figure 2:**
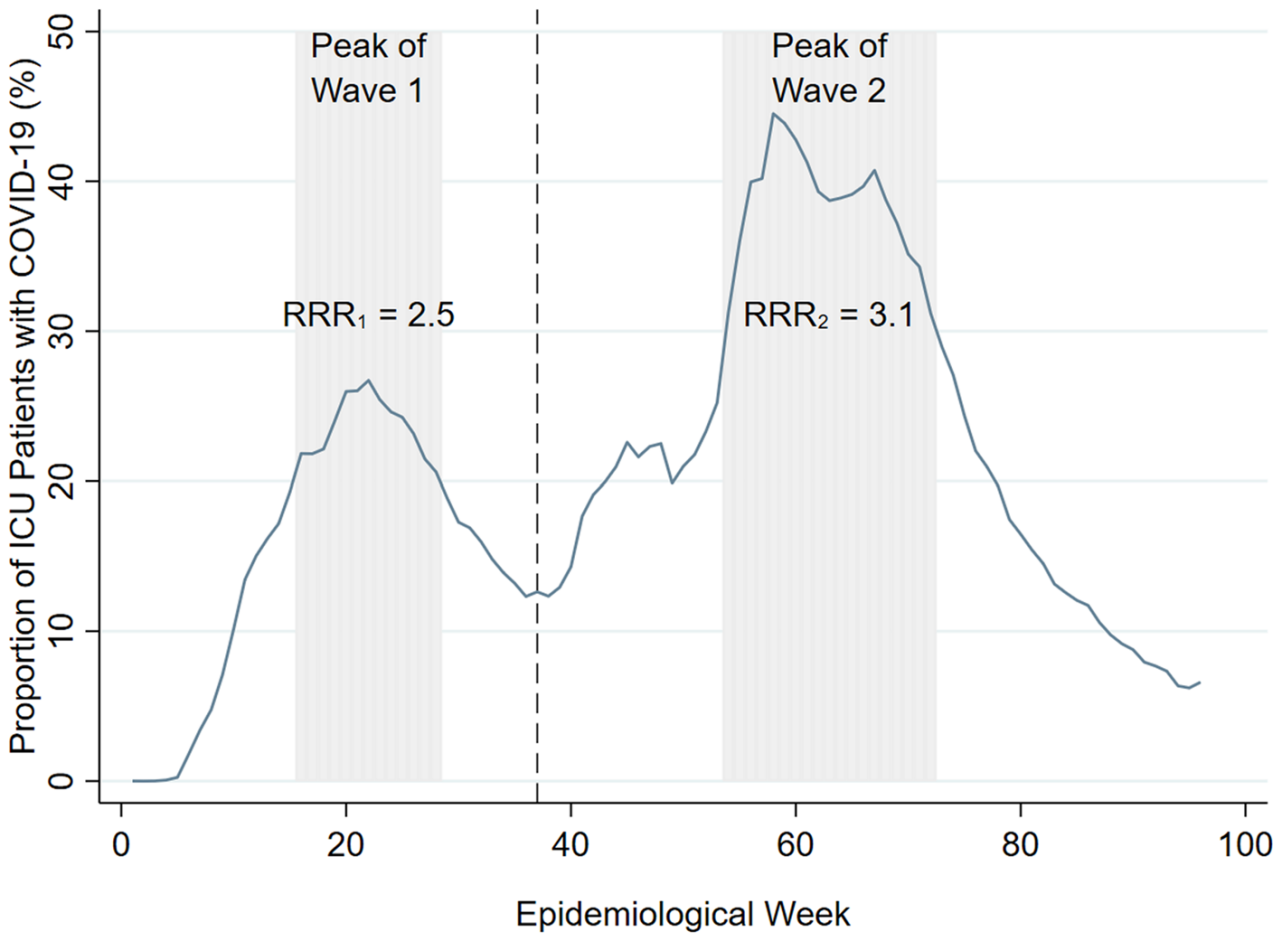
Evolution of the average proportion of COVID-19 ICU patients over 96 epidemiological weeks (from February 26, 2020 to December 31, 2021). The vertical line at week 37 represents the demarcation between Brazil’s first and second COVID-19 waves. The ${RRR}_{1}$ represents the relative spillover mortality risk ratio during the first wave, i.e. ${RRR}_{1}= \frac{Risk \,\left(Peak \; Wave 1\right)}{Risk \,(Off{\text{-}}peak \; Wave 1)}=\frac{2.86\%}{1.15\%}=2.5 (95\% \mathrm{CI} \,1.95{-}3.05)$. For the second wave, ${RRR}_{2}= \frac{Risk\, \left(Peak \;Wave 2\right)}{Risk \,(Off{\text{-}}peak \; Wave 2)}=\frac{1.58\%}{0.51\%}=3.1 (95\% \mathrm{CI} \,1.29{-}4.95)$.

The results above show that variations in the hospital-week share of COVID-19 ICU patients generate variations in the mortality risk of non-COVID-19 ICU patients. Figure 2 plots the COVID-19 ICU share across time. Following Zeiser et al., the figure splits the 96 epidemiological weeks into two periods: first and second waves of COVID-19, which are separated on November 5, 2020 (week 37)^26^. The shaded regions represent periods of high hospitalization rates of COVID-19 patients in each wave. For the first wave, the peak region corresponds to weeks 16–28 when the average proportion of COVID-19 ICU patients was above 20%. For the second wave, the peak corresponds to weeks 54–72 when the share of COVID-19 patients was above 30%. Using wave-specific data, we estimate a variation of the high-dimensional fixed effect panel regression that differentiates between peak and off-peak spillover mortality risks (refer to supplemental materials Section 3 for details). This allows us to estimate the spillover mortality risk ratio for a non-COVID-19 patient receiving intensive care in a peak week relative to an off-peak week.

During the first wave, the relative risk ratio of peak spillover risk against off-peak spillover risk is 2.5 (95% CI 1.95–3.05). That is, the additional mortality risk imposed by COVID patients on non-COVID patients is 2.5 times higher in a peak week relative to an off-peak weak. For the second wave, the relative risk ratio increases to 3.1 (95% CI 1.29–4.95).

## Discussion

The COVID-19 pandemic imposed difficult challenges to health care facilities that were tasked to handle the influx of both COVID and non-COVID patients. As more data becomes available, it is clear that the pandemic led not only to a crisis of COVID-19 patients, but to a crisis of healthcare systems as a whole. There is increasing evidence suggesting that non-COVID-19 sectors of health care have been affected by the pandemic. Examples include elective surgical procedures^27,28^, spinal pathology^29^, cancer care^30^, Kawasaki disease^31,32^, malaria prevention activities^33^, and organ transplants^34^.

A growing literature compares pre- and post-pandemic in-hospital mortality rates of non-COVID patients. After controlling for a number of observable characteristics, papers in this literature report post vs pre-pandemic odds ratio of 1.20 (95% CI 1.19–1.21) in the United States^14^, 1.65 (95% CI 1.53–1.79) in Italy^35^, and 1.28 (95% CI 1.23–1.32) in Denmark^15^. In Brazil, some papers use SUS pre-pandemic data to predict in-hospital mortality rate in 2020. With a focus on non-COVID-19 respiratory disease, Albuquerque et al. find that the actual mortality during the pandemic (12.89%) is significantly higher than predicted mortality rate (8.23%, p < 0.005)^36^. Normando et al. find that the mortality rate of patients with cardiovascular diseases during the pandemic (11.52%) is significantly higher than that predicted by the pre-pandemic trend (10.39%, pvalue < 0.05)^37^. Using data from a private hospital network in Brazil, a study compares 2020 ICU mortality rates against those from 2019. The authors report statistically significant odds ratios from 1.189 (95% CI 1.091–1.296) for patients with SAPS3 equal to 70, to 1.421 (95% CI 1.269–1.591) for patients with SAPS3 equal to 20^12^.

In contrast with the above literature, we focus on variations in mortality risk during the pandemic. A study in the United States finds that the proportion of patients hospitalized for COVID-19 is correlated with their in-hospital mortality rate^38^. We use similar pandemic data to examine ICU mortality of non-COVID patients. Using a large dataset and panel data methods to control for unobservable confounders, our paper offers new insights by showing that hospital-week variation in the proportion of COVID-19 ICU patients influences the mortality risk of critically ill non-COVID-19 patients. That is, the results show that the effects of the pandemic spill over into the population of non-COVID-19 patients. The estimates of spillover mortality risks (summarized in Fig. 1) suggest that the death toll of the COVID-19 pandemic is significantly higher than that coming from those that succumb to COVID-19. Applying the average spillover of 1.3% to SUS population of non-COVID ICU patients, i.e. 1,477,653 patients, our results suggest that 19,209 non-COVID ICU deaths can be attributed to the pandemic. To put this number in perspective, note that it represents 30.8% of the difference between the WHO’s calculations of excess deaths and reported COVID-19 deaths in Brazil^39^.

The paper has several limitations. First, while we focus on the mortality of ICU patients, it is also possible for the pandemic to impact both mortality and morbidity of non-ICU non-COVID-19 patients. Such impacts deserve more attention and health care delivery would benefit from rigorous empirical analyses using large datasets. Second, the paper restricts the analysis to patients in need of intensive care. However, it is possible for pandemic spillovers to exist in the population of non-ICU patients.

Third, our variable that measures the local intensity of the pandemic, the ICU-level proportion of COVID-19 patients, serves as a reduced form proxy for a variety of factors in the sense that the number of COVID-19 patients is correlated with health demand and supply drivers. That is, the stronger is the pandemic, the greater is the potential for a more fragile health status of non-COVID-19 patients due to treatment delays, the more unfavorable is the doctor-patient ratio, the more strained is the health infrastructure of the system, the more rigid are hospital protocols, all of which can impose difficult challenges to delivering health care to non-COVID-19 patients. While we pose such hypotheses, our data do not allow us to test for specific mechanisms. Future research is needed to uncover mechanisms responsible for the spillover effects reported in the paper.

Fourth, while our models control for hospital fixed effects, data limitations prevent us from controlling for an important time-varying hospital characteristic: hospital occupancy level, or patients per bed. During the pandemic, Brazil struggled to keep up with the demand for COVID-19 intensive care beds^40^. It is reasonable to expect that, in most cases, possible SUS expansion in ICU beds likely targeted COVID-19 units. If the number of beds available to non-COVID ICU patients were stable in the timeframe of our analysis, then the specification of hospital fixed effects along with the number of patients controls for the unobserved effect of hospital occupancy level. This important subject should be the target of additional research.

Finally, robustness of our estimates rely on the assumption that patients are correctly classified into COVID and non-COVID groups. It is possible for measurement errors to exist and some of the non-COVID patients in our data might in fact be undiagnosed COVID patients, especially those with “Respiratory System” diseases. To the extent that these errors exist, they may lead to an overestimation of spillover effects since we would then incorrectly include COVID deaths as part of non-COVID mortality. This is, nevertheless, unlikely to affect a significant portion of our sample since patients in ICU settings are highly monitored.

## Methods

### Data

Technological advances and their economic feasibility have increased healthcare providers’ ability to store and process large amounts of data, contributing to an increase in the adoption of electronic administrative records^41^. Big data has become a new healthcare ally making a wide range of contributions from resource allocation to diseases diagnosis^42^. In health, big data is often defined as electronic records that provide data volume (large databases), velocity (high speed of access), variety (data heterogeneity), and veracity (quality and reliability)^43–45^.

In this paper, we leverage information from large datasets of SUS electronic administration records. SUS databases are maintained by the SUS Information Technology department, namely DATASUS. The databases are anonymized and contain no individual identifiers. The data are made publicly available by DATASUS, which contributes to data velocity as information is promptly accessible online for research and analyses. All methods were carried out in accordance with relevant guidelines and regulations.

The key database used in this work is the Hospital Information System (SIH/SUS). The SIH maintains records of all care provided during hospitalizations financed by SUS in either public or private hospitals (with contracts to offer free health care under the SUS system)^46^. The database contains a large volume of data and catalogs the universe (i.e. millions) of hospitalizations in SUS. Hospitalization records of closely monitored ICU patients are produced in the hospital through a system called Hospital Admission Authorization (AIH/RD). The existence of a single system for all SUS hospitals ensures that clinically relevant information is stored in a unified format. In addition, intensive care is one of the most costly healthcare services, which provides incentives for hospitals to keep good records to avoid complications related to funding, payments, and reimbursements. All of these factors contribute to the veracity of the data.

The AIH/RD contains a variety of information. The medical information contains key characteristics of the hospitalization, including unique national hospital code and hospital location, date and length of stay, reason for hospitalization (ICD-10 code^47^), whether or not intensive care was utilized, and if yes, type of ICU bed (COVID-19 or non-COVID-19), and hospitalization outcome (e.g. discharged, transferred, or deceased). In addition, since socioeconomic context of patients affects their health status, the database also records information about patients’ place of residence (postal code), along with sex and age.

### Sampling

The sample is drawn from the universe of all patients hospitalized in the SUS network. The time period of the analysis starts on February 26, 2020 (date of the first COVID-19 ICU hospitalization in Brazil) to December 31, 2021, corresponding to 96 epidemiological weeks. In this time window, SUS offered intensive care to 2,006,387 patients. Our data consists of concluded treatments (no censoring) such that outcomes are measured at the end of the hospital stay. It is also important to distinguish patients by COVID-19 status. COVID-19 patients are identified based on the ICD-10 diagnosis code B342 or by type of ICU, i.e. patients in ICU beds designated for treatment of COVID-19 infection.

Our focus is the mortality of non-COVID-19 patients in intensive care units. As a result, in constructing the dependent variable of our regression model, we drop 528,734 COVID-19 ICU patients. Note that COVID-19 ICU patients are used to construct the primary independent variable: the proportion of COVID-19 patients. In addition, we drop 136 patients classified under chapter 22 of the ICD-10, namely ‘codes for special purposes’, because this chapter is used for the provisional assignment of new diseases and it is possible that some of these patients could be infected with the novel SARS-CoV-2 virus. Moreover, since our focus is within hospital spillover effects (from COVID-19 to non-COVID-19), we drop 99,022 non-COVID-19 ICU patients in hospitals that did not hospitalize a COVID-19 patient in the sampling period. We also drop 3077 non-COVID-19 patients that according to the records were hospitalized in field hospitals or new ICUs (i.e. those that start functioning after the start of the pandemic). It is possible that these observations reflect extraneous conditions, where patients that tested negative for COVID-19 were admitted in field hospitals due to capacity issues in the system or may simply represent data errors.

Finally, to establish a link between within hospital spillover effects and health outcomes, we focus on patients that finished their hospitalization in the same hospital where spillover effects are measured, i.e. we focus on patients that were either discharged or died. As such, we drop 62,137 non-COVID-19 ICU patients that were transferred to other hospitals. Finally, we exclude four observations with data errors in the ICD-10 disease code.

Our final sample consists of a large dataset with 1,313,277 non-COVID-19 ICU patients, which contributes to the external validity of our findings. The dataset captures significant heterogeneity by collecting information from patients in all regions of Brazil. Specifically, patients in the sample are residents of 21,518 (5-digit level) postal codes in 5567 (out of 5570) municipalities of Brazil. These patients received intensive care in 989 hospitals, in 473 cities, with 1508 different principal diagnoses (ICD-10 3-digit code). These statistics show that this study is amongst the largest and most comprehensive examinations to date of impacts of the COVID-19 pandemic on intensive care patients.

Average mortality risk of non-COVID-19 ICU patients is 22.3%. Average age is 47.55 and 44.6% of patients are female. On average, the total number of (COVID-19 and non-COVID-19) ICU patients in a hospital-week cell is 40.5, of which 13.8% are diagnosed with COVID-19. Approximately one quarter of the non-COVID-19 ICU patients in the sample received intensive care due to ‘diseases of the circulatory system (ICD-10 codes I00-I99), 11% due to ‘injury, poisoning and certain other consequences of external causes’ (S00-T98), 10.7% due to ‘certain conditions originating in the perinatal period’ (P00-P96), 10.4% due to ‘diseases of the respiratory system (J00-J99), 9.8% due to ‘certain infectious and parasitic diseases’ (ICD-10 codes A00-B99), 9% due to ‘neoplasms’ (C00-D48), 6.6% due to ‘diseases of the digestive system’ (K00-K93), and the reasons for ICU admission of the remaining 17% of patients are distributed amongst the remaining 13 chapters (see Table 1, supplemental materials).

### Estimation model

We are interested in estimating the impact COVID-19 ICU hospitalization on outcomes of patients whose intensive care is not associated with a SARS-CoV-2 infection. We focus on the ICU mortality risk of non-COVID-19 patients as the health outcome $(Y)$. Next, we develop a variable to measure the COVID-19 spillover risk faced by a non-COVID-19 ICU patient. For each hospital-week, we divide the population of ICU patients into two groups: COVID-19 and non-COVID-19. We use the proportion of ICU patients reported to be infected with the COVID-19 virus $(P)$ as a measure of how severe the demand for COVID-19 intensive care is at a given hospital-week data cell. We argue that, if our empirical model reveals that an increase in the proportion $P$ leads to an increase in the mortality of non-COVID-19 patients $Y$, then the model offers empirical evidence of spillover effects in the form of *spillover mortality risk*, i.e. additional mortality risk that a non-COVID-19 ICU patient faces for being treated in a hospital-week with a proportion $P$ of COVID-19 patients.

To identify spillover mortality risk, our methods must account for several sources of unobserved heterogeneity that can result in confounding effects and bias the estimates of spillover risks. Our empirical strategy leverages information from high granularity patient-level microdata by employing high-dimensional panel data estimation methods^48–51^. We test for spillover effects by estimating the following regression model with four high-dimensional fixed effects:

1 $${Y}_{ipjtd}=f\left({P}_{jt}\right)+g\left({X}_{ipjtd}\right)+\beta {Z}_{jt}+ {\mu }_{p}+{\gamma }_{j}+ {\delta }_{t}+ {\rho }_{d}+ {\varepsilon }_{ipjtd}$$

where $Y$ is a binary death indicator for non-COVID-19 ICU patient $i$, with residence in the postal code $p$, receiving intensive care in hospital $j$, at epidemiological week $t$, with main diagnosis given by the IDC-10 disease code $d$. ${P}_{jt}$ is the proportion of ICU patients in hospital $j$ and epidemiological week $t$ diagnosed with COVID-19. In order to allow for nonlinear spillover effects, we specify $f\left({P}_{jt}\right)$ as a cubic polynomial function. The term $g\left(X\right)$ specifies a cubic polynomial to capture sex and sex-specific nonlinear age affects^24^. The model controls for the size of the hospital by including $Z$, the total number of ICU patients in hospital $j$ and week $t$.This is important because heavy hospital-load can increase in-hospital mortality^25^. The error term is $\varepsilon$ while $\mu$, $\gamma$, $\delta$, and $\rho$ are high-dimensional fixed effects.

The model’s fixed effects serve the fundamental role of controlling for unobserved heterogeneity. ${\mu }_{p}$ is a postal code fixed effect. The availability of more than 21 thousand postal codes offers a remarkable opportunity for absorbing socioeconomic components of health demand. While administrative medical records offer detailed health-related information, typically this type of data lack variables that describe socioeconomic determinants of health such as income and education. This poses a challenge for studies that use administrative records because the strength of the local COVID-19 epidemic is correlated with low socioeconomic status^16^. In fact, pandemics are particularly challenging precisely because they exploit pre-existing inequalities in health care systems^17^. For example, regions of Brazil with high risk of COVID-19 outbreaks are also regions with the most socially vulnerable patients^18^. Therefore, studies that do not rigorously control for health demand will fail to identify pandemic impacts because the strength of the pandemic is strongly correlated with socioeconomic characteristics and resulting estimates will be biased by confounding effects. We leverage the postal code information contained in SIH/SUS records to control for demand determinants. By including a high-dimensional postal code fixed effect, our empirical model is able to estimate spillover effects holding constant local socioeconomic conditions of patients, where local is defined in a highly granular manner (21,518 five-digit postal codes). This is especially powerful in our application because Brazil is a country with strong inequalities and social segregation^52,53^.

The term ${\gamma }_{j}$ represents a hospital fixed effect. This component absorbs unobserved determinants of health outcomes that are fixed at the hospital level. This term improves identification by capturing influences driven by type of hospital, fixed protocols, management and overall hospital quality, or any other hospital characteristic that has not varied in the short-run (time period of the analysis). This is important because estimates of impacts on the health outcomes can significantly change when differences between hospitals that are fixed over time are accounted for^19^. The epidemiological week fixed effect ${\delta }_{t}$ captures shocks that are common to all hospitals and patients in the system, including changes in national public policy such as availability of vaccines and national-level COVID-19 testing capacity, as well as the evolution of national infection waves^20,21^. Finally, ${\rho }_{d}$ captures disease-specific unobserved determinants of mortality and allow us to identify spillover effects isolating both demand and supply factors associated with each one of the 1508 ICD-10 diseases leading to intensive care of patients in our sample. Controlling for disease diagnosis is important as mortality rates vary by ICD code^22,23^.

With over a million observations and four high-dimensionality fixed effects, estimation of our empirical model poses a significant computational burden. We estimate the model using Sergio Correia’s feasible estimator for high-dimensional fixed effects, which has significant advantages in processing times compared to alternative approaches^48,54^. We also allow observations to be correlated at the hospital level and implement inference based on cluster-robust standard errors with hospital clustering^55^. Model (1) is estimated using Stata 17. Coefficient estimates are reported in Table 2 (supplemental materials).

### Supplementary Information

Supplementary Information.

## Data Availability

The paper uses publicly available data from the Brazilian Unified Health System (SUS). The data is from SUS’s administrative records that are anonymized to protect patient identity. For more information refer to https://datasus.saude.gov.br/acesso-a-informacao/. All data generated and/or analysed during the current study are available in the GitHub repository, https://github.com/brunowichmann/non-COVID-19-ICU-patients.

## Code availability

All analyses reported in this study used the statistical software Stata 17. All Stata files are available publicly at https://github.com/brunowichmann/non-COVID-19-ICU-patients.
