# Supplementary material for: Big data evidence of the impact of COVID-19 hospitalizations on mortality rates of non-COVID-19 critically ill patients
Source: Sci Rep. 2023 Aug 21;13:13613. doi: 10.1038/s41598-023-40727-z (PMC10442321; doi:10.1038/s41598-023-40727-z)
Supplement: Supplementary file 1 — Supplementary Information. [file 41598_2023_40727_MOESM1_ESM.pdf]

Supplemental Materials:

Big data evidence of the impact of COVID-19 hospitalizations on mortality rates of non-COVID-19 critically ill patients

Journal: Scientific Reports. DOI: 10.1038/s41598-023-40727-z

Bruno Wichmann*
University of Alberta, Edmonton, Canada.

Roberta Moreira Wichmann
World Bank, Brasília, Brazil
&
Brazilian Institute of Education, Development and Research – IDP, Brasília, Brazil.

* Corresponding author. Department of Resource Economics & Environmental Sociology.
College of Natural and Applied Sciences. University of Alberta.
503 General Services Building. Edmonton, AB, Canada. T6G-2H1.
Phone: +1 (780) 492-0819. Fax: (780) 492-0268.
Email: bwichmann@ualberta.ca

1. Summary Statistics

Table 1: Descriptive Statistics of non-COVID-19 ICU patients (N = 1,313,277)

Variable	Description	Mean	Std. Dev.
Mortality	Outcome of patient at the end of ICU hospitalization. =1 if patient died, 0 otherwise	0.223	0.416
Age	Age of patient (years)	47.55	27.95
Female	=1 if patient is female, 0 male	0.446	0.497
No. of patients[(a)]	Total number of ICU patients (includes COVID-19 and non-COVID-19)	40.51	41.31
COVID proportion[(a)]	Proportion of ICU patients with COVID-19 diagnosis (in decimal notation)	0.138	0.183
Disease Classification[(b)]			
Ch 1	Certain infectious and parasitic diseases (A00-B99)	0.098	0.297
Ch 2	Neoplasms (C00-D48)	0.090	0.286
Ch 3	Diseases of the blood and blood-forming organs and certain disorders involving the immune mechanism (D50-D89)	0.005	0.068
Ch 4	Endocrine, nutritional and metabolic diseases (E00-E90)	0.017	0.130
Ch 5	Mental and behavioural disorders (F00-F99)	0.001	0.029
Ch 6	Diseases of the nervous system (G00-G99)	0.027	0.161
Ch 7	Diseases of the eye and adnexa (H00-H59)	0.000	0.020
Ch 8	Diseases of the ear and mastoid process (H60-H95)	0.000	0.012
Ch 9	Diseases of the circulatory system (I00-I99)	0.255	0.436
Ch 10	Diseases of the respiratory system (J00-J99)	0.104	0.306
Ch 11	Diseases of the digestive system (K00-K93)	0.066	0.249
Ch 12	Diseases of the skin and subcutaneous tissue (L00-L99)	0.007	0.084

Ch 13	Diseases of the musculoskeletal system and connective tissue (M00-M99)	0.009	0.093
Ch 14	Diseases of the genitourinary system (N00-N99)	0.037	0.189
Ch 15	Pregnancy, childbirth and the puerperium (O00-O99)	0.017	0.130
Ch 16	Certain conditions originating in the perinatal period (P00-P96)	0.107	0.309
Ch 17	Congenital malformations, deformations and chromosomal abnormalities (Q00-Q99)	0.020	0.139
Ch 18	Symptoms, signs and abnormal clinical and laboratory findings, not elsewhere classified (R00-R99)	0.024	0.154
Ch 19	Injury, poisoning and certain other consequences of external causes (S00-T98)	0.110	0.313
Ch 21	Factors influencing health status and contact with health services (Z00-Z99)	0.006	0.077

Notes: (a) Variable is measured at the hospital level, and at week of patient's outcome. (b) Refer to https://icd.who.int/browse10/2019/en for more information related to the International Statistical Classification of Diseases and Related Health Problems 10th Revision. ICD-10 codes in parenthesis.

2. Estimates of the high-dimensional fixed effects mortality, model (1)

Table 2 shows the coefficient estimates of model (1). The first three coefficients are used to calculate estimates of the spillover mortality risk:

$$\widehat{f(P_{jt})} = 0.08820P_{jt} - 0.13765{P_{jt}}^2 + 0.17754{P_{jt}}^3$$

where P_{jt} denotes the proportion of ICU patients with COVID-19 diagnosis (Proportion COVID, measured in decimal terms). Similar calculations can be made for the risk associated with the age of males (using the coefficients of age, age^2, and age^3) or females (using the coefficients of female*age, female*age^2, and female*age^3). The table also shows that, after controlling for sex-specific age patterns (and other factors in the model), the mortality risk of female non-COVID-19 ICU patients is 0.73 percentage points higher than that of males (p<0.01). The table also shows larger hospitals have lower mortality rates. Specifically, the mortality risk of a non-COVID-19 ICU patient decreases, on average, by 0.079 percentage points per additional ICU

patient (p<0.01). Finally, the baseline mortality risk is 5.281%. This baseline is the mortality risk free from the effect of COVID patients (spillover), age and sex, number of patients, as well as hospital, epidemiological week, patient's postal code, and patient's disease fixed effects.

Table 2: high-dimensional fixed effect model estimation results

Proportion COVID	0.08820***
	(0.01707)
Proportion COVID^2	-0.13765**
	(0.06170)
Proportion COVID^3	0.17754***
	(0.06009)
Age	0.00492***
	(0.00042)
Age^2	-0.00004***
	(0.00001)
Age^3	0.0000004***
	(0.0000001)
Female	0.00730***
	(0.00157)
Female*Age	-0.00190***
	(0.00024)
Female*Age^2	0.00006***
	(0.00001)
Female*Age^3	-0.0000004***
	(0.00000005)
No. of Patients	-0.00079***
	(0.00012)
Constant	0.05281***
	(0.00688)
F-stat (11, 987)	460.61
R-squared	0.2087
N	1,311,866

*Notes: Dependent variable is mortality of non-COVID-19 ICU patients. Robust standard errors clustered at the hospital level are reported in parentheses. The high-dimensional regression model absorbs hospital, epidemiological week, patient's postal code, and patient's disease fixed effects. Regression drops 1,411 singleton observations. * p<0.10 ** p<0.05 ***p<0.01.*

3. Estimates of peak and off-peak relative risk ratios, by COVID-19 wave (Figure 2).

Figure 2 plots the COVID-19 ICU share across time. Throughout the study period, the average hospital-week has less than 50% of COVID ICU patients. Since the spillover mortality risk is fairly linear up to 50% of COVID-19 patients (see figure 1 Panel C), the time shape of figure 2 is very similar to that of figure 1 panel B.

Following Zeiser et al., the figure splits the sample into two groups based on the timing of hospitalizations.[1] Hospitalizations before November 5, 2020 (week 37) are denoted first wave hospitalizations, while those after are second wave hospitalizations. In each wave, we further divide observations into two subgroups: peak and off-peak. For the first wave, we denote as 'peak' weeks with average COVID-19 ICU shares above 20% of patients, which correspond to epidemiological weeks 16-28. For the second wave, 'peak' denotes weeks with average COVID-19 ICU shares above 30% of patients, which correspond to epidemiological weeks 54-72. This design is summarized in the table 3.

Table 3: Sample splitting - first and second waves, peak and off-peak observations

	First Wave	Second Wave
Timeframe	Weeks 1 – 36<br>(Feb 26, 2020 – Nov 3, 2020)	Weeks 38 – 96<br>(Nov 11, 2020 – Dec 31, 2021)
Peak	Weeks 16 – 28	Weeks 54 – 72
Off-peak	Weeks 1–15 and 29–36	Weeks 38–53 and 73–96

Spillover risks for each COVID-19 wave are obtained by wave-specific observations to estimate the following high-dimensional fixed effects model:

$$Y_{ipjtd} = f(P_{jt}) + peak\, f(P_{jt}) + g(X_{ipjtd}) + \beta Z_{jt} + \mu_p + \gamma_j + \delta_t + \rho_d + \varepsilon_{ipjtd}, \quad (2)$$

where $peak$ is a binary indicator for observations in epidemiological weeks with peak infection rates. Coefficient estimates are available in table 4.

Table 4: Wave-specific estimates of high-dimensional fixed effect, model (2)

	(1)<br>First Wave	(2)<br>Second Wave
Proportion COVID	0.19563***	0.04649*
	(0.03541)	(0.02493)
Proportion COVID^2	-0.43084***	-0.03197
	(0.14167)	(0.09498)
Proportion COVID^3	0.39978***	0.07700
	(0.14234)	(0.09672)
Peak*Proportion COVID	-0.08779*	-0.01955
	(0.04480)	(0.03278)
Peak*Proportion COVID^2	0.19442	0.06231
	(0.18201)	(0.12057)
Peak*Proportion COVID^3	-0.10150	-0.03541
	(0.17858)	(0.11721)
Age	0.00471***	0.00499***
	(0.00048)	(0.00044)
Age^2	-0.00003***	-0.00005***
	(0.00001)	(0.00001)
Age^3	0.0000004***	0.0000005***
	(0.0000001)	(0.0000001)
Female	0.00587**	0.00781***
	(0.00242)	(0.00190)
Female*Age	-0.00138***	-0.00217***
	(0.00035)	(0.00030)
Female*Age^2	0.00004***	0.00006***
	(0.00001)	(0.00001)
Female*Age^3	-0.0000003***	-0.0000005***
	(0.0000001)	(0.0000001)
No. of Patients	-0.00085***	-0.00075***
	(0.00015)	(0.00013)
Constant	0.05553***	0.05210***
	(0.00807)	(0.00717)
F-stat (14, 982)	282.82	328.07
R-squared	0.2308	0.2161
N	482,204	813,713

*Notes: In both regressions, the dependent variable is mortality of non-COVID-19 ICU patients. The model in column (1) uses only observations from the first COVID-19 wave (epidemiological weeks 1-36). The model in column (2) uses only observations from the second COVID-19 wave (epidemiological weeks 38-96). Robust standard errors clustered at the hospital level are reported in parentheses. Both models employ high-dimensional fixed effects that absorb hospital, epidemiological week, patient's postal code, and patient's disease effects. Regression (1) drops 1,839 singleton observations. Regression (2) drops 1,585 singleton observations. * p<0.10 ** p<0.05 ***p<0.01.*

The estimates of the average relative risk ratios of peak vs off-peak spillover mortality reported in Figure 2 are computed as:

$$RRR = \frac{\text{Spillover Risk in Peak Weeks}}{\text{Spillover Risk in Offpeak Weeks}} = \frac{\sum_{k=1}^{3}(\widehat{\alpha_k} + \widehat{\beta_k})P_p^k}{\sum_{k=1}^{3}\widehat{\alpha_k}\, P_o^k}$$

where $\widehat{\alpha_k}$ (for $k = 1,2,3$) represent the coefficients of Proportion COVID, Proportion COVID^2, and Proportion COVID^3, and $\widehat{\beta_k}$ (for $k = 1,2,3$) represent the coefficients of Peak*Proportion COVID, Peak*Proportion COVID^2, and Peak*Proportion COVID^3 (see table 4). P_p represents the average across non-COVID patients of the proportion of COVID-19 ICU patients during peak weeks, while P_o represents the corresponding off-peak average.

4. Institutional Background

This section offers a brief overview of Brazil's public health care system, the Unified Health System (from Portuguese: *Sistema Único de Saúde* -- SUS). Refer to the work of Paim *et. al.* and Castro *et. al.* for a deeper understanding of SUS, its history, and general health trends in Brazil [2,3].

SUS was established in 1988 to meet the constitutional right that every citizen should have access to health care, free of charge, co-payments, or deductibles. SUS carries out about 2.8 billion visits per year, from simple outpatient visits to highly complex procedures such as organ transplantation.[4] In addition to consultations, exams and hospitalizations, SUS also implements immunization campaigns, epidemiological, environmental and pharmaceutical assistance, as well as health surveillance actions, e.g. food inspections and drug registration [5]. Over the years, SUS developed a far-reaching primary care network that has a proven record of successfully implementing large-scale vaccination programs [6].

The implementation of SUS also contributes to the reformulation of the Brazilian health planning strategy that created a more systematic and long-term national healthcare development policy. Prevention and health promotion actions were included in the planning of the national health policy, in addition to curative services, contributing to the formulation of a systemic approach to health in Brazil.

To produce meaningful results in a large geographical area, SUS is designed as a decentralized system where health care programs rely on the coordination between federal and local governments. The supply of SUS services is organized in a hierarchical and regionalized manner. Resources are distributed based on the level of medical care complexity, with higher complexity services centralized under higher level (e.g. federal). Service allocation planning is carried out regionally following epidemiological criteria designed to optimize health care delivery. SUS management is also decentralized and each level of government - federal, state, and municipal - is responsible for the control and supervision of health care in a way that is also correlated with the complexity of the services provided. For example, the municipalities are responsible for primary care, while the states and the federal government offer high and medium complexity services.

SUS faces challenges that are proportional to the mission of offering comprehensive and universal care in a country with more than 215 million inhabitants. Among the major challenges are problems related to management and underfunding of the system, as evidenced by the growth of expenses in detriment of revenue [7]. Such problems could compromise the quality of care and/or lead to interruption of service provision. While private health services are available in Brazil, only a small fraction of the population has the resources to finance private medical care.

Despite the challenges, SUS has contributed to the reduction of hospitalization and mortality by reducing health access inequalities in Brazil. Many health indicators suggest that SUS contributed to the democratization of access to health services in the country and SUS utilization rate is higher among the most vulnerable individuals. The variation in the coverage rate reflects socioeconomic background. According to recent data, the lower the education and income, the greater the dependence on the public health network for medical and dental care [8].

In Brazil, the reach of its public health system's network made some experts believe that the country would be in a relatively favourable position to manage the COVID-19 pandemic [9]. However, since the first confirmed case in February 2020, Brazil has struggled to develop and coordinate interventions to control the spread of the COVID-19 virus, including challenges with the implementation of a vaccination campaign that initially heavily relied on the less effective CoronaVac vaccine [10–12].

5. References

1. Zeiser, F. A. *et al.* First and second COVID-19 waves in Brazil: A cross-sectional study of patients' characteristics related to hospitalization and in-hospital mortality. *The Lancet Regional Health - Americas* **6**, (2022).
2. Paim, J., Travassos, C., Almeida, C., Bahia, L. & MacInko, J. The Brazilian health system: History, advances, and challenges. *The Lancet* Preprint at https://doi.org/10.1016/S0140-6736(11)60054-8 (2011).
3. Castro, M. C. *et al.* Brazil's unified health system: the first 30 years and prospects for the future. *The Lancet* Preprint at https://doi.org/10.1016/S0140-6736(19)31243-7 (2019).
4. Oswaldo Cruz Foundation. Pense SUS. https://pensesus.fiocruz.br/sus (2022).
5. Brazilian Ministry of Health. Sistema Único de Saúde. http://tabnet.datasus.gov.br/cgi/sih/Proced_hosp_loc_int_2008.pdf (2022).
6. Barreto, M. L. *et al.* Successes and failures in the control of infectious diseases in Brazil: Social and environmental context, policies, interventions, and research needs. *The Lancet* Preprint at https://doi.org/10.1016/S0140-6736(11)60202-X (2011).

7. Fernandes, G. A. de A. L. & Pereira, B. L. S. The challenges of funding the brazilian health system in fighting the covid-19 pandemic in the context of the federative pact. *Revista de Administracao Publica* (2020) doi:10.1590/0034-761220200290x.

8. Brazilian Institute of Geography and Statistics. PNS 2019: sete em cada dez pessoas que procuram o mesmo serviço de saúde vão à rede pública. https://agenciadenoticias.ibge.gov.br/agencia-sala-de-imprensa/2013-agencia-de-noticias/releases/28793-pns-2019-sete-em-cada-dez-pessoas-que-procuram-o-mesmo-servico-de-saude-vao-a-rede-publica (2020).

9. Castro, M. C. *et al.* Spatiotemporal pattern of COVID-19 spread in Brazil. *Science (1979)* (2021) doi:10.1126/science.abh1558.

10. Candido, D. S. *et al.* Evolution and epidemic spread of SARS-CoV-2 in Brazil. *Science (1979)* (2020) doi:10.1126/SCIENCE.ABD2161.

11. Mallapaty, S. China COVID vaccine reports mixed results — what does that mean for the pandemic? *Nature* (2021) doi:10.1038/d41586-021-00094-z.

12. Ranzani, O. T. *et al.* Effectiveness of the CoronaVac vaccine in older adults during a gamma variant associated epidemic of covid-19 in Brazil: test negative case-control study. *theBMJ* **374**, (2021).
